# Drinking water supplemented with wood vinegar on growth performance, intestinal morphology, and gut microbial of broiler chickens

**DOI:** 10.14202/vetworld.2021.92-96

**Published:** 2021-01-12

**Authors:** Kornkamon Hanchai, Tassanee Trairatapiwan, Rachakris Lertpatarakomol

**Affiliations:** 1 Department of Clinical Veterinary Science, Faculty of Veterinary Medicine, Mahanakorn University of Technology, 140 Cheum Samphan Road, Nong Chok, Bangkok, 10530 Thailand; 2 Department of Animal Science and Veterinary Basic Science, Faculty of Veterinary Medicine, Mahanakorn University of Technology, 140 Cheum Samphan Road, Nong Chok, Bangkok, 10530 Thailand

**Keywords:** broiler, growth performance, gut microbial, intestinal morphology, wood vinegar

## Abstract

**Background and Aim:**

Wood vinegar is a product generated from the combustion and distillation of wood and other plant materials. It has been shown to suppress bacteria, resulting in healthier livestock and increased yields. This study aimed to determine the efficacy of drinking water supplemented with wood vinegar on growth performance, intestinal health, and gut microbial of broilers.

**Materials and Methods:**

A total of 120 Ross 308 1-day-old male broiler chicks were randomly distributed in a completely randomized experimental design. The study consisted of three treatments containing four replicates, with 10 birds in each. Treatments were given 0.5% and 1.0% (V/V) wood vinegar supplemented in drinking water, while no supplementation was given to the control group. The animals were raised in an open-house system. All groups were provided with a commercial diet and drinking water *ad libitum*. Analysis of variance was conducted using the general linear model procedure to compare the levels of wood vinegar supplementation in drinking water on growth performance, intestinal morphology, and gut microbial.

**Results:**

No significant differences (p>0.05) were found for body weight gain, feed intake, feed conversion ratio, and water consumption between groups during the starter (1-21 days old), grower (22-35 days old), and whole (1-35 days old) growth periods. Moreover, no significant differences in villi height and crypt depth (p>0.05) at 21 and 35 days of age were found. In addition, no significant difference in terms of lactic acid bacteria and *Escherichia coli* was found between the different treatments.

**Conclusion:**

Drinking water supplemented with wood vinegar was not found to have an effect on the growth performance and gut microbial of broiler chickens in the present study. However, the supplementation of wood vinegar in drinking water could improve intestinal morphology.

## Introduction

Continual use of antibiotics as growth promoters in modern livestock production encourages the retention of drug residues in animal tissues, and human consumption of such animal products could potentially increase antibiotic resistance processes [1]. These issues have led to regulatory and consumer acceptance pressures concerning the need to ban the use of antibiotics in animal production [2]. Organic acids, short-chain fatty acids, are considered to be a potential growth promoter alternative to antibiotics for animals [3-5].

Wood vinegar or pyroligneous acid is a by-product of charcoal production and is generated from the combustion and distillation of wood and other plant materials. Wood vinegar is a complex mixture of 80-90% water and 10-20% organic compounds. The major component of wood vinegar is acetic acid and it also contains various phenolic compounds such as guaiacol and cresol, as well as organic acids including acetic, formic, and propionic acids [6,7]. The key objective of dietary acidification is to inhibit gut bacterial competing with the host for available nutrients and reducing the metabolite of possible toxic bacteria [8,9]. In addition, wood vinegar is also used to suppress bacteria, resulting in healthier livestock and increasing yields [7], while it has also been shown to remove odor-causing agents from animals’ bodies and waste [10]. The previous studies report that the provision of wood vinegar supplementation has several advantageous effects indicating improvements to both the general health of poultry and swine, in addition to growth and feed efficiency [11-13]. Nonetheless, wood vinegar can be obtained from a variety of raw material sources and can contain different organic components.

In liquid form, wood vinegar can easily be used as a supplement for small-scale farmers who provide their animals with commercial feed. The present study, therefore, aimed to evaluate the efficacy of wood vinegar supplementation in drinking water on growth performance, intestinal morphology, and gut microbial in broiler chickens.

## Materials and Methods

### Ethical approval

The experimental procedure was approved by the Animal Care and Use Committee, Mahanakorn University of Technology.

### Study period and location

The present study was conducted from December 2018 to May 2019 and undertaken at the Poultry Research Station of Faculty of Veterinary Medicine, Mahanakorn University of Technology, Thailand.

### Bird management and experimental design

A total of 120 Ross 308 1-day-old male broiler chicks with an average bodyweight of 46±2.6 g were obtained from a commercial hatchery. The animals were raised in an open-house system and were randomly distributed in a completely randomized experimental design (completely randomized design) into three groups containing four replicates with 10 birds in each. Each pen had a floor space of 1×1 m^2^ space and the floor was covered with rice hull.

The experimental treatments consisted of: (1) Chicks fed with fresh water (0.0% wood vinegar) as the control group; (2) chicks fed with 0.5% (V/V) wood vinegar in drinking water; and (3) chicks fed with 1.0% (V/V) wood vinegar in drinking water. The wood vinegar utilized in the present study was made from *Myristica fragrans* and *Acacia confuse* produced by the Royal Agricultural Station, Angkhang, Thailand. Wood vinegar was added to the drinking water twice daily (9:00 and 17:00). Feedings were given to the animals *ad libitum* with two commercial diet formulas for starter (1-21 days old) and grower (22-35 days old) growth stages. The chickens had free access to drinking water. Medication and vaccinations were administered. The daily water consumption of the chicks in each replicate was recorded. Body weight (BW) and feed intake (FI) were recorded at 21 and 35 days of age according to the feed formula (starter and grower diets). BW gain (BWG) and feed conversion ratio (FCR) were then calculated.

### Examination of intestinal morphological structure

At 21 and 35 days of age, two birds from each replicate were randomly dissected to collect small intestinal samples. The middle of jejunum samples was sectioned horizontally in 2 cm lengths [14]. All samples were fixed in 10% formalin. After fixation, the samples were dehydrated by increasing the concentration of alcohol and embedded in paraffin. Embedded tissue samples were sectioned at a 5 mm thickness using an auto-microtome (Leica RM 2155, Leica Microsystems GmbH, Nussloch, Germany). The slides were stained with hematoxylin and eosin. The villus height and crypt depth were determined in cross-section using Axiolab inverted microscope (Carl Zeiss, Hamburg, Germany) equipped with an HBO 50 camera to capture images and subsequently analyzed using Zen 2012 (blue edition) software (Carl Zeiss Microscopy GmbH, Hamburg, Germany). The measurements were examined on 10 villi and 10 crypts for each segment. The villus height-to-crypt depth ratios were then calculated.

### Quantitative examination of gut microbial

At 35 days of age, the cecum of two chicks from each replicate was collected. Ceca contents were used to determine the amount of lactic acid bacteria according to the procedure of ISO 15214:1998 and *Escherichia coli* in accordance with the procedure of AOAC (2016) 991.14.

### Statistical analysis

Analysis of variance was conducted using a general linear model command of SPSS version 17.0 (SPSS Inc., Chicago, IL, USA) to compare the levels of wood vinegar supplementation in drinking water on growth performance, intestinal morphology, and gut microbial. Duncan’s new multiple range test was used to make comparisons between groups. Values of p≤0.05 were considered to be statistically significant.

## Results and Discussion

### Growth performance

The data is shown in Table-1 and indicates no significant difference (p>0.05) in water consumption among the treatments, despite increased concentrations of wood vinegar resulting in a pH reduction of the drinking water. The supplementation of wood vinegar in drinking water did not influence (p>0.5) BWG, FI, or FCR during the starter (1-21 days old), grower (22-35 days old), and whole (1-35 days old) growth periods.

**Table-1:** Effect of drinking water supplemented with wood vinegar on growth performance of broiler chickens.

Parameters	Wood vinegar			SEM	p-value

	0.0%	0.5%	1.0%		
pH in drinking water	6.0	5.0	4.0	N/A	N/A
1-21 days of age					
Water consumption (mL/bird)	2954.86	3142.42	2949.95	61.55	0.38
BWG (g/bird)	595.98	594.90	586.28	9.06	0.91
FI (g/bird)	900.50	902.37	848.58	11.93	0.10
FCR	1.51	1.52	1.45	0.02	0.10
22-35 days of age					
Water consumption (mL/bird)	4360.74	5105.06	4264.71	202.20	0.18
BWG (g/bird)	1091.44	1171.10	1073.87	21.67	0.15
FI (g/bird)	1495.09	1596.03	1437.75	8.336	0.15
FCR	1.37	1.36	1.34	0.01	0.60
1-35 days of age					
Water consumption (mL/bird)	7315.60	8247.48	7214.66	248.42	0.18
BWG (g/bird)	1687.42	1766.00	1660.15	26.03	0.24
FI (g/bird)	2395.59	2498.41	2286.32	39.85	0.08
FCR	1.42	1.41	1.38	0.01	0.21

BWG=Body weight gain, FI=Feed intake, FCR=Feed conversion ratio, SEM=Standard error of mean. N/A=Not analyzed

The results obtained from this research are similar to Ruangwittayanusorn *et al*. [15] who concluded that the supplementation of wood vinegar in diets showed no adverse effects on growth performance of broilers (p>0.05). Another study [11] reported that during the starter periods (1-10 days of age), supplementation with wood vinegar in drinking water improves the feed efficiency of broiler chickens. However, it did not significantly affect (p>0.05) BWG, FI, and FCR for the whole growth periods (1-42 days of age). Furthermore, the previous research indicates that supplementing drinking water with organic acids has no effect on BWG and FCR (p>0.05) of broiler chickens [16]. In contrast, some studies have found positive effects from the supplementation of wood vinegar or organic acids in broilers [4], laying hens [5], quails [13], weaning pigs [7], and growing pigs [12]. These positive effects may influence by the general characteristics of organic compounds, such as improvement of the gastrointestinal tract, enhancement of nutrient digestibility, and the competitive elimination of pathogenic bacteria [17]. The reason for not showing the positive effects of supplementing drinking water with wood vinegar on growth parameters in the present study could be due to the proper conditions of rearing and feeding broiler chickens [18].

### Intestinal morphology

As presented in Table-2, there was no significant difference (p>0.05) in villi height and crypt depth among the different groups. At 21 days of age, chicks in the control group (0.0%) had higher (p<0.05) villus: Crypt than those fed with 0.5% and 1.0% wood vinegar in drinking water, but there was no significant difference (p>0.05) at 35 days of age. Nonetheless, the results obtained from this research showed that wood vinegar added to drinking water led to an improved intestinal morphology, although the values were not significant. Chicks that drank water containing 0.5% and 1.0% wood vinegar had higher villi and thicker crypt than those which drank the non-supplemented water.

**Table-2:** Effect of drinking water supplemented with wood vinegar on intestinal morphology of broiler chickens.

Parameters	Wood vinegar			SEM	p-value

	0.0%	0.5%	1.0%		
21 days of age					
Villi height (µm)	801.21	855.28	803.41	23.88	0.62
Crypt depth (µm)	120.08	142.79	149.03	6.06	0.11
Villus:crypt	7.07^a^	6.16^b^	5.78^b^	0.22	0.02
35 days of age					
Villi height (µm)	903.90	1063.15	1034.94	34.90	0.13
Crypt depth (µm)	138.41	144.52	145.66	8.76	0.95
Villus:crypt	7.22	7.98	7.69	0.34	0.70

^a,b^Means in the same row with different superscripts are significantly different (p≤0.05)

The results of a previous study indicate that wood vinegar improved intestinal morphology characteristics, especially in terms of increasing villi height and crypt depth among broilers [11]. An increased length of intestinal villi results in improved functions of secretion, digestion, and nutrient absorption [17,19]. Moreover, increasing villi height and crypt depth correlate with increased epithelial cell turnover [9,20]. Thicker crypts demonstrate fast tissue turnover to promote regeneration of the villus as required in response to mucosal stripping or inflammation from pathogens or their toxins [21]. Consequently, the use of organic acid reduced intestinal colonization and infection, thereby suppressing inflammatory emergence at the intestinal mucosa [17].

### Gut microbial

The results obtained from the present study indicate no significant difference (p>0.05) on cecal microbial (lactic acid bacteria and *E. coli*) between treatments (Table-3). Similar results report that diets supplemented with raw and distilled wood vinegar did not affect the number of coliform, salmonella, and lactic acid bacteria in excreta and intestine. However, 4% dietary distilled wood vinegar increased lactic acid bacteria in ileum [22]. It is understood that organic acids have a strong effect on pathogenic bacteria and leads to decreased colonization [3]. The undissociated form of organic acids can pass through the bacteria cell membrane. Further, the acid breaks down in alkaline medium of bacterial cytoplasm to produce H^+^ ions which lower the cell pH. The ­organism uses its energy to restore normal cell equilibrium, whereas the RCOO^-^ anions produced from the acid can interfere DNA and protein synthesis. On events occurring, the organism is under stress and unable to replicate rapidly [9,17]. In addition to its antimicrobial property, it also possesses the ability to lower the pH of the gastrointestinal tract and increase the number of lactic acid bacteria [22,23]. The components and quantity of organic components were not classified in the wood vinegar utilized in the present study, so it appears to be highly difficult to completely clear the effect of gut microbial, as seen in the present study and in others. However, this could properly be the concentration of wood vinegar supplementation which was too low.

**Table-3:** Effect of drinking water supplemented with wood vinegar on gut microbial of broiler chickens.

Parameters	Wood vinegar			SEM	p-value

	0.0%	0.5%	1.0%		
Lactic acid bacteria (log cfu/g)	7.60	6.99	7.29	0.23	0.60
*Escherichia coli* (log cfu/g)	6.68	7.17	6.42	0.19	0.26

## Conclusion

The present study revealed that drinking water supplemented with 0.5% and 1.0% wood vinegar had no effect on growth performance and gut microbial of broiler chickens. Nonetheless, the results suggest that supplementing drinking water with wood vinegar could improve intestinal morphology. In addition, the findings from this preliminary study will be utilized in a future study along with higher levels of wood vinegar supplementation.

## Authors’ Contributions

KH, TT, and RL compiled the research ideas and designed the main framework. KH and RL composed the manuscript. KH and TT contributed in coordinating field data collections. TT and RL conducted the statistical analysis. RL compiled and revised the manuscript. All authors have read and approved the final manuscript.
